# Lifelong Learning-Enabled Fractional Order-Convolutional Encoder Model for Open-Circuit Fault Diagnosis of Power Converters Under Multi-Conditions

**DOI:** 10.3390/s25061884

**Published:** 2025-03-18

**Authors:** Tao Li, Enyu Wang, Jun Yang

**Affiliations:** 1 College of Railway Transportation, Hunan University of Technology, Zhuzhou 412007, China; 2 College of Mechanical and Vehicle Engineering, Hunan University, Changsha 410082, China; 3 Zhuzhou Times New Material Technology Co., Ltd., Zhuzhou 412007, China; yangjun@csrzic.com; 4 College of Electrical and Information Engineering, Hunan University of Technology, Zhuzhou 412007, China; m22085800032@stu.hut.edu.cn

**Keywords:** lifelong learning, power converter, open-circuit fault, fault diagnosis, fractional order

## Abstract

Open-circuit (OC) faults in power converters are common issues in motor drive systems, significantly affecting the safe and stable operation of the system. Conventional models can accurately diagnose faults under a single operating condition. However, when conditions change, these models may fail to recognize new fault features, resulting in a decrease in diagnosis accuracy. To address this challenge, this paper proposes a lifelong learning-enabled fractional order-convolutional encoder model for open-circuit fault diagnosis of power converters under multi-conditions. Firstly, the model automatically extracts and identifies fault signal features using the convolutional module and the encoder module, respectively. Subsequently, the model’s iterative computational process is optimized by learning historical gradient information through fractional order, and enhancing the model’s ability to capture the long-term dependencies inherent in fault signals. Finally, a multilevel lifelong learning framework has been established to enable the model to continuously learn the fault features of power converter under multi-conditions, thereby avoiding catastrophic forgetting that can occur when the model learns different tasks. The proposed model effectively addresses the challenge of low fault diagnosis accuracy that occurs when the operating conditions of the power converter change, achieving a diagnosis accuracy of 96.89% across 85 fault categories under multi-conditions.

## 1. Introduction

The power converter, as a core component of motor drive systems, is widely used in fields such as electric vehicles, renewable energy generation, and rail transportation. During operation, the power converter often needs to switch frequently between the rectifier and inverter states, which may lead to faults under multi-conditions. Therefore, the ability to quickly and accurately diagnose faults in power converters across multi-conditions is essential for ensuring the safe operation of motor drive systems.

Power converter faults are primarily classified into short-circuit (SC) faults and open-circuit (OC) faults, which are manifested by the failure of internal power devices such as an insulated gate bipolar transistor (IGBT) [1]. The main causes of SC faults can be divided into two aspects. On one hand, abnormal Pulse Width Modulation (PWM) control signals may lead to SC faults. On the other hand, IGBT breakdown may occur under high-stress conditions such as over-voltage, over-current, or excessive junction temperature. When an SC fault occurs, protective devices within the power converter, such as circuit breakers or fast-acting fuses, are activated to isolate the failed component and shut down the power converter [2]. The primary cause of OC faults is IGBT thermal fatigue failure, in which failure of internal solder layers or bonding wires prevents the IGBT from conducting current [3]. Once an OC fault occurs, the output current of the power converter becomes distorted and unbalanced; this does not trigger protective devices but severely reduces the output power quality and may even lead to secondary faults [4]. This paper focuses on the diagnosis of OC faults in the power converter.

For OC faults, current diagnosis methods mainly include model-based methods [5,6,7], signal-based methods [8,9,10], and data-driven methods [11,12,13]. Model-based methods require the establishment of mathematical models that characterize the physical behavior of the actual system. By observing and calculating the residuals between the measured values of the actual system and the simulated values from the mathematical model, the current state of the system can be assessed. In reference [14], the current residual is calculated by comparing the actual current path with the estimated current path, and faults are identified based on characteristic residual patterns. In reference [15], the extended Kalman filter is adjusted by measuring currents on both the battery and capacitor sides until the residual falls below a set correction threshold, and fault detection and localization are achieved by determining whether the maximum correction count or correction values on the faulted side exceed the threshold. In reference [16], output current and its rate of change are used as fault detection variables, while the sum of phase current and neutral point voltage residuals is used for fault localization. In reference [17], a process of state augmentation and nonsingular coordinate transformation was designed for the observation system to address misdiagnosis issues arising from interactions between different fault types. Model-based methods generally offer high diagnosis accuracy; however, establishing mathematical models for complex systems is challenging. Furthermore, when system parameters change, the model needs to be redefined.

An IGBT OC fault leads to voltage oscillations at the load end of the power converter and introduces significant harmonics in the output current. Fault diagnosis methods based on observing these signal variations are referred to as signal-based methods. When an OC fault occurs in the IGBT, the current path in its corresponding bridge arm changes. Reference [18] proposes diagnosing inverter OC faults by utilizing changes in the current path under effective vector coordinates. Reference [19] designed a normalized cost function for detecting OC faults and used the current mean and phase angle for fault localization. In addition to current detection, voltage signals can also be used to diagnose IGBT OC faults. Reference [20] utilizes the similarity of capacitor voltages under fault and normal conditions in modular multilevel converters, employing a correlation coefficient for early-stage fault localization. Reference [21] performs fault diagnosis based on the characteristic that the common-mode voltage of an inverter remains equal during normal operation and reduces fault miss-detection rates by injecting active common-mode voltage. Reference [22] proposed a fault diagnosis method based on the dynamic characteristics of midpoint voltage and a fault-tolerant control strategy based on Complementary Switch Blocking, which mitigates fault impacts by blocking gate drive signals of the complementary switch. Reference [23] processes three-phase voltage signals using ensemble empirical mode decomposition, breaking them down into intrinsic mode functions and calculating their norm entropy to characterize fault signal statistics. However, in practical applications, current signals are susceptible to load effects, reducing diagnosis accuracy and increasing diagnosis time. Voltage-based methods often require additional sensors, raising operational and maintenance costs.

Data-driven methods build fault diagnosis models by training on large amounts of historical data to establish a mapping relationship between fault features and fault modes. Once the model detects abnormal data, it can quickly identify the fault type using previously learned knowledge, completing the diagnosis process. For online fault diagnosis applications, the model needs to respond rapidly to fault signals. Numerous studies have aimed to improve diagnosis speed and efficiency to meet real-time requirements. For IGBT OC faults in three-phase PWM inverters, Reference [24] designed an ensemble classifier based on the Extreme Learning Machine with a reliability mechanism and used a Random Vector Functional Link (RVFL) network to identify fault features, reducing diagnosis time. Building on this, reference [25] proposed an RVFL network based on the Fast Fourier Transform and Relief algorithm to prevent misdiagnosis due to similar fault features. Reference [26] introduced a diagnosis method for IGBT OC faults in Neutral Point Clamped (NPC) three-level inverters, using Independent Component Analysis and Joint Approximate Diagonalization algorithms to separate fault signals, allowing for more precise identification of complex fault patterns. Reference [27] proposed a fault detection and localization method based on the Entropy of Wavelet Packets feature extraction and Support Vector Machine (SVM) to diagnose IGBT OC faults in multilevel inverters. Reference [28] considered the multi-scale characteristics of fault signals and developed a fault diagnosis algorithm based on multi-scale Approximate Entropy, Dempster–Shafer evidence theory, and Deng entropy fusion, effectively addressing conflicts and uncertainties among features and enhancing the mutual benefits of features across different scales.

The aforementioned methods demonstrate rapid processing speeds and high accuracy in diagnosing OC faults, they still rely on feature extraction algorithms to derive various characteristics from the raw signals. The diagnosis efficacy of these methods is significantly constrained by the selection process of features based on individual expert experience. This human intervention not only adds to operational complexity but may also introduce the risk of misjudgment. In contrast, deep neural networks possess exceptional feature parsing and selection capabilities, allowing them to automatically identify and select optimal fault features based on internal signal correlations, thereby enhancing the accuracy and reliability of fault diagnosis [29,30]. Reference [31] proposed a deep convolutional network model based on the inception module, which can automatically extract and identify fault features in three-phase PWM converters, applicable in both inverter and rectifier states. Reference [32] eliminated redundant features and sampling points through correlation analysis, followed by wavelet transformation to further compress feature data, accelerating the training process of deep feedforward networks. Reference [33] combined neural network models with classification algorithms, achieving rapid fault feature identification along the time dimension using an improved Long Short-Term Memory (LSTM) network, while employing SVM to classify the outputs of the enhanced LSTM, thus addressing the interpretability issues of neural network outputs. Reference [34] introduced a wide residual network model based on incremental learning, which combines the automatic feature extraction capability of residual networks with the incremental learning ability of generalized learning systems, allowing for incremental learning of new data without retraining the model. To tackle the challenges of extracting fault features from NPC inverters under non-stationary conditions, reference [35] proposed a fault diagnosis method based on attention collaborative stacked LSTM (ASLSTM) networks, which extracts highly discriminative features from multi-source time series data, improving the stability of fault diagnosis for NPC inverters.

All of the aforementioned models diagnose OC faults in power converters by analyzing current or voltage signals. However, in practice, these signals are usually affected by the electromagnetic environment and produce burrs, spikes, and other disturbances, which may reduce the diagnostic accuracy of the models. Fractional order gradient descent is an optimization method based on fractional order calculus, which allows for the incorporation of historical information during the gradient update, thereby enhancing the model’s learning capability. Compared to traditional integer-order gradient descent, fractional order gradient descent can adjust the optimization path of the neural network model through non-integer-order gradients, improve global search capabilities, and thus enhance the model’s robustness against noise. Reference [36] proposed an optimizer based on fractional order momentum gradient descent, which enables the neural network model to better converge to the global optimal solution, and improves the fault diagnosis accuracy under small sample datasets. Reference [37] employed the long-term memory property of Caputo–Fabrizio fractional order derivatives to solve the local dependence and singularity problems of traditional integer orders in practical applications, and improved the extraction effect of weak fault signals. Reference [38] utilizes the fractional order chaotic system to map the original vibration signals into the three-dimensional chaotic space, constructs a 3D dynamic error phase map with obvious differentiation ability, and realizes the rapid classification of fault signals.

Although neural networks exhibit high accuracy in fault diagnosis, when the operating state of the power converter changes, the neural network model needs to be retrained on new fault features, which leads to a decrease in diagnosis accuracy. Therefore, this paper proposes a lifelong learning-enabled fractional order-convolutional encoder model that can continuously learn the open-circuit fault characteristics of the power converter under multi-conditions. This model maintains high diagnostic accuracy for all faults, highlighting its significant research implications and practical applications. The contributions of this paper are as follows:

1. A convolutional encoder model was constructed for learning and diagnosing OC faults in the power converter. The time series features of three-phase current fault signals and the relative positional relationships between each phase signal are automatically extracted by the convolutional module. The encoder module is utilized to identify and classify these features, enabling the automatic learning and diagnosis of fault samples.

2. Fractional order is utilized to optimize the convolutional encoder model. This model improves the optimization process of backpropagation by incorporating fractional order, allowing it to fully consider the historical gradient information when updating, and is conducive to capturing long-term dependencies in time-series data. Additionally, the smooth gradient update path reduces the oscillations during the optimization process, facilitating stable convergence to the global optimal solution and improving robustness against anomalous noise.

3. A multilevel lifelong learning framework is designed to enable the model to continuously learn from new fault samples. Limit the range of updates during which the model parameters learn new tasks by incorporating a resilient regularization penalty term into the loss function. In addition, a random small number of samples from previous tasks are inserted into the new fault samples and by learning the soft labels from earlier tasks to improve the stability and accuracy of the model under different tasks.

## 2. Fault Analysis

**Figure 1:**
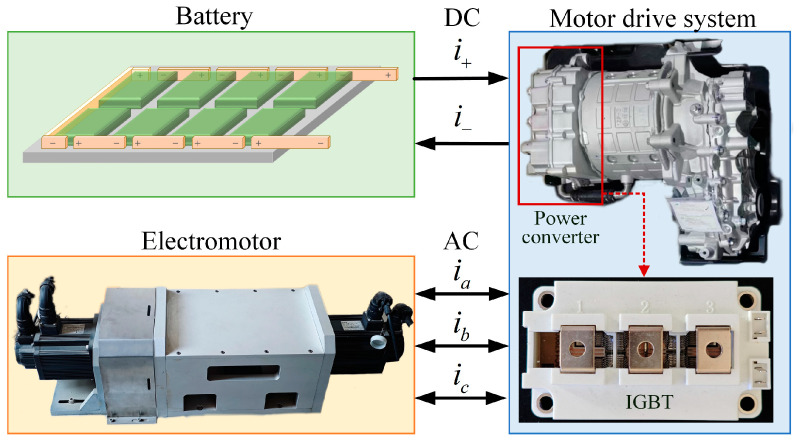
Motor drive system structure.

The structure of the motor drive system is shown in Figure 1, consisting mainly of a battery, power converter, and motor. When the system operates the motor, the power converter works in the inverter state, converting the electric energy from direct current (DC) to alternating current (AC) and delivering it to the motor. Conversely, when the system is in a braking state, the motor operates in generator mode, outputting three-phase AC to the power converter. At this time, the power converter works in a rectifier state, converting the three-phase AC into DC and storing it in the battery. Therefore, during the operation of the motor drive system, the power converter continuously alternates between rectifier and inverter state.

**Figure 2:**
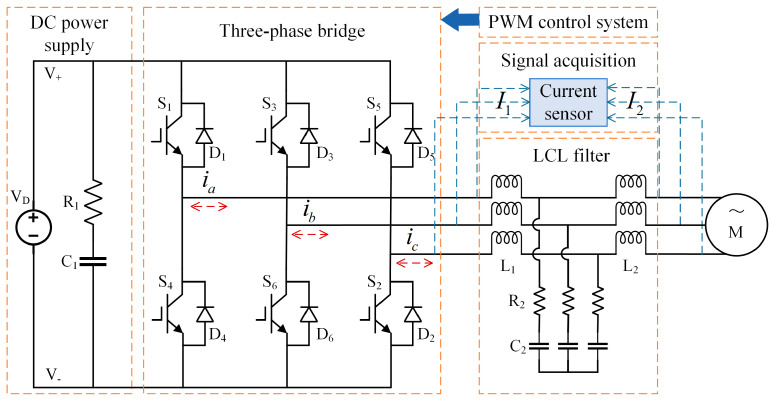
Power converter circuit diagram.

The circuit structure of the power converter is shown in Figure 2. The DC power source *V_D_*, capacitor *C*_1_, and resistor *R*_1_ form an equivalent DC power source responsible for supplying and storing electrical energy. *S_x_* and *D_x_* (*x* = 1, 2, …, 6) represent IGBT and Fast Recovery Diode (FRD), respectively. The three-phase bridge circuit consists of six pairs of IGBT with anti-parallel FRD. The three-phase AC, *i_k_* (*k* = *a*, *b*, *c*), flows through the LCL filter—comprising an inductor-capacitor-inductor configuration—before being supplied to the motor. The LCL filter comprises inductors *L*_1_ and *L*_2_, resistor *R*_2_, and capacitor *C*_2_. The six pairs of IGBT in the three-phase bridge circuit are turned on and off in a specific pattern by the PWM control system, enabling bidirectional energy conversion between AC and DC. The signal acquisition module captures the system’s three-phase currents using current sensors. In rectification mode, the sensor captures the power converter’s bridge arm current *I*_1_ as the fault diagnosis signal, and in inverter mode, it captures the load-side current *I*_2_. An IGBT OC fault causes them to remain off, independent of PWM control, leading to current distortions that vary with converter operating conditions and motor types, such as AC Induction Motors (ACIM) or Permanent Magnet Synchronous Machines (PMSMs) in electric vehicles. This study thus examines the approach for diagnosing IGBT OC faults in the power converter under multi-conditions and motors.

In practical applications, diagnosing single or double OC faults is more practical as the probability of multiple OC faults occurring simultaneously is very low. This helps to identify and locate the failed device in time, preventing the fault from spreading to other parts of the system, which could result in secondary failures or even system paralysis. Furthermore, the OC fault diagnosis approach proposed in this paper is not restricted to a specific circuit topology or a particular model of IGBT device. This is because the approach can automatically learn and recognize fault characteristics from the signal. And no matter which topology or device, the occurrence of an OC fault will produce distinct abnormal features in the current or voltage signal. Consequently, the approach is universal and can be extended to new power converters with redundant designs or more switching devices, thus providing theoretical support and technical guarantee to improve the safety and reliability of the overall system. Based on the multi-conditions of the power converter and various motors, four distinct operating tasks can be classified, as shown in Table 1.

The simulation circuit is constructed using the MATLAB/Simulink R2022b platform according to the circuit topology of Figure 2 to simulate the current waveforms of the power converter when OC failure occurs under different tasks, and the parameters of the simulation circuit are shown in Table 2. The MATLAB/Simulink R2022b software is developed by MathWorks Incorporated (Natick, MA, USA).

### 2.1. Fault Analysis of the Power Converter Working Under Inverter Condition

When the power converter operates in the inverter state, energy transfers from the DC to the AC side. During normal operation, the output currents *i_a_*, *i_b_*, and *i_c_* are balanced three-phase currents. To examine the waveform changes, phase A current *i_a_* is used as an example. In the first half of the *i_a_* cycle, *S*_4_ remains off while *S*_1_, controlled by the PWM controller, alternates on and off in a set pattern, outputting a PWM square wave voltage with amplitude *V_D_* and positive polarity to phase A. This PWM square wave voltage, acting on an inductive load, produces a sinusoidal current *i_a_* in phase A, where *i_a_* > 0. In the second half-cycle, the pattern is reversed, with *S*_1_ off and *S*_4_ controlled by PWM, resulting in a sinusoidal current *i_a_* with negative polarity.

**Figure 3:**
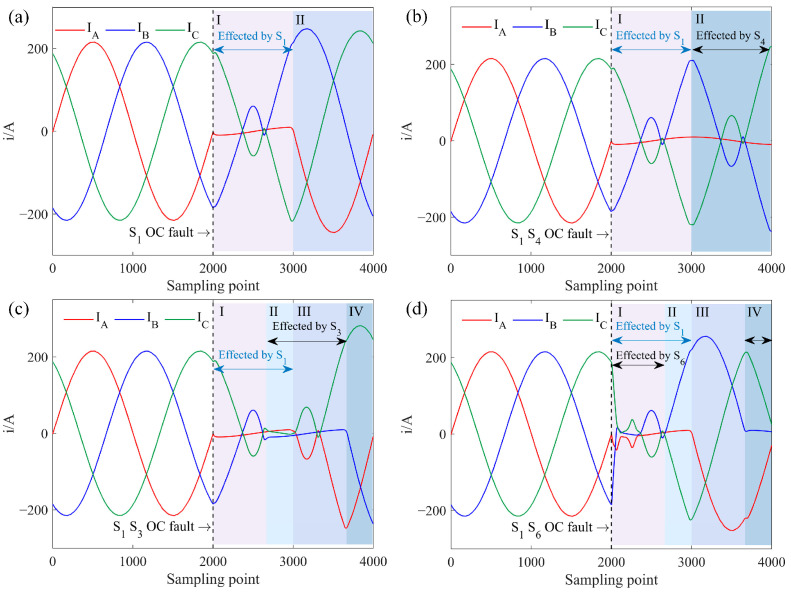
Three-phase current simulated waveform of IGBT OC fault under Task A, where I–IV represent different segments of the waveform. (**a**) *S*_1_ OC fault. (**b**) *S*_1_ *S*_4_ OC fault. (**c**) *S*_1_ *S*_3_ OC fault. (**d**) *S*_1_ *S*_6_ OC fault.

For Task A, with an ACIM, the three-phase current simulated waveform with an OC fault in *S*_1_ is shown in Figure 3a. In the second cycle, when *S*_1_ fails, it is effectively off, and with *S*_1_ also off, the DC side voltage cannot reach the AC side through phase A, leading to *i_a_* = 0 in the first half-cycle. The resulting unbalanced power among the three phases distorts the currents in the non-faulted phases *i_b_* and *i_c_*, causing harmonic generation and amplitude increase. If *S*_4_ experiences an OC fault, there will be no current output in the second half of *i_a_*. Similar current waveform changes occur in phases B and C with single IGBT OC faults.

Dual IGBT OC faults can occur in three configurations: (1) faults in the same-phase IGBTs, (2) faults in different-phase IGBTs in the same half-bridge, and (3) faults in different-phase IGBTs in different half-bridges. Figure 3b shows the three-phase current simulated waveform with OC faults in *S*_1_ and *S*_4_, representing same-phase faults. Since *S*1 and *S*_4_ affect separate halves of the *i_a_* cycle independently, the fault waveform appears as a superposition of two symmetrical single IGBT faults in phase A, resulting in zero current in phase A while currents *i_b_* and *i_c_* remain opposite.

Figure 3c illustrates the three-phase current simulated waveform when *S*_1_ and *S*_3_ experience faults, representing different-phase faults within the same half-bridge. In the three-phase bridge circuit, any two phases in the same half-bridge have a 120^◦^ phase difference. Since each phase IGBT conducts over a 180^◦^ interval, overlapping occurs when a dual OC fault happens in the same half-bridge. Based on the distinct distortions, the three-phase current waveform is divided into four segments. Segment I is affected only by *S*_1_, and Segment III only by *S*_3_, behaving as single IGBT faults. Segment II shows overlapping effects from *S*_1_ and *S*_3_ faults, with both phases A and B OC, and *i_a_* = *i_b_* = *i_c_* = 0 due to the three-phase current vector sum equaling zero. Segment IV remains unaffected by faults.

*S*_1_ and *S*_6_ represent faults in different phases and half-bridges, with a 120^◦^ phase overlap. Their dual fault three-phase current simulated waveform is shown in Figure 3d. Segments II and IV are influenced by individual IGBT faults, while Segment III remains fault-free. Segment I reflects overlapping effects from both *S*_1_ and *S*_6_ faults, resulting in *i_a_* = 0 and *i_b_* = −*i_c_*. Due to the increased current magnitude in phases B and C, the rapid current drop induces intense oscillations and distortion.

**Figure 4:**
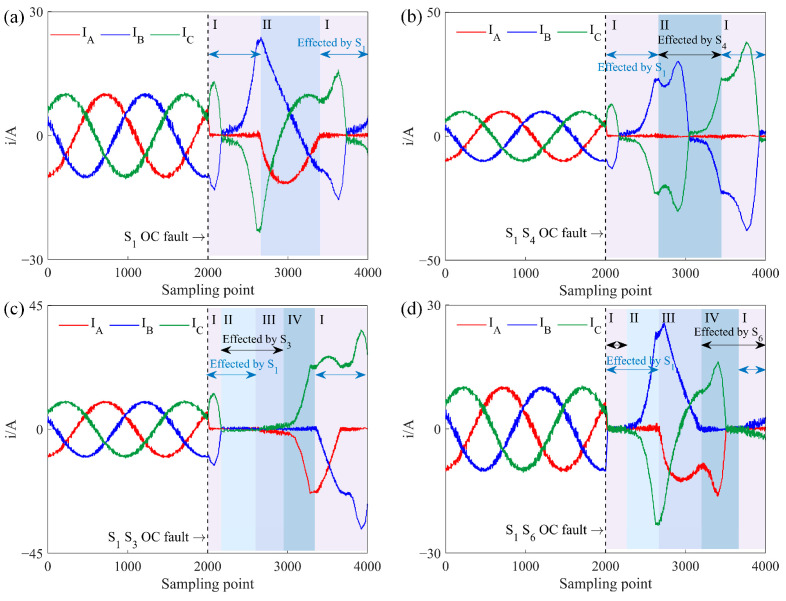
Three-phase current simulated waveform of IGBT OC fault under Task B, where I–IV represent different segments of the waveform. (**a**) *S*_1_ OC fault. (**b**) *S*_1_ *S*_4_ OC fault. (**c**) *S*_1_ *S*_3_ OC fault. (**d**) *S*_1_ *S*_6_ OC fault.

For Task C, with a PMSM, a typical three-phase current simulated waveform under an OC fault is shown in Figure 4. Similarly to the previous analysis, a single IGBT OC fault results in no current in the affected phase for half of the cycle, while the currents in the other phases become distorted. Figure 4a illustrates the three-phase current simulated waveform with an OC fault in *S*_1_. In cases of dual IGBT OC faults, the analysis depends on whether there is an overlap in the faulted phases. Non-overlapping segments behave as single IGBT faults, while overlapping segments follow the same analysis as Task A. However, due to the differences in motor type and control strategy, the current distortions resulting from IGBT faults vary compared to other tasks.

### 2.2. Fault Analysis of Power Converter Working Under Rectifier Condition

**Figure 5:**
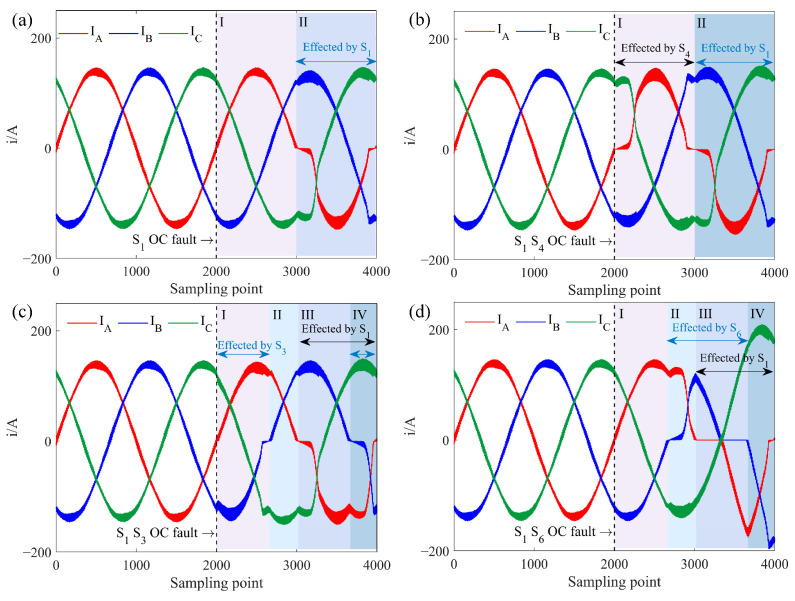
Three-phase current simulated waveform of IGBT OC fault under Task C, where I–IV represent different segments of the waveform. (**a**) *S*_1_ OC fault. (**b**) *S*_1_ *S*_4_ OC fault. (**c**) *S*_1_ *S*_3_ OC fault. (**d**) *S*_1_ *S*_6_ OC fault.

When the power converter operates in a rectifier state, energy flows from the AC to the DC side. For Task B, where the motor is an ACIM, a typical three-phase current simulated waveform under an OC fault is shown in Figure 5. When the rectifier is operating normally, symmetrical three-phase voltage at the AC side generates symmetrical three-phase current through the load inductance.

Taking A-phase current *i_a_* as an example, the three-phase current simulated waveform under a single IGBT OC fault is analyzed. When *i_a_* > 0, the current flows through *D*_1_ to the DC side or returns to the AC side through *S*_4_. When *i_a_* < 0, the current flows back to the AC side via either *S*_1_ or *D*_4_. Thus, an OC fault in *S*_1_ affects only the *i_a_* < 0 part, as shown in Figure 5a, where *i_a_* = 0 during this period due to the lack of return current through *S*_1_. When the A-phase voltage reaches the lowest value among the three phases, *D*_4_ conducts to carry the current back to the motor. In the latter half-cycle of *i_a_*, two no-current intervals appear since *D*_4_ turns off when other diodes are conducting. Due to the constant vector sum of the three-phase currents, some distortion also occurs in *i_b_* and *i_c_*.

The types of dual IGBT OC faults in a rectifier state are similar to those in an inverter state. Figure 5b shows the three-phase current simulated waveform for an OC fault in the A-phase IGBT, where only *D*_1_ and *D*_4_ conduct, causing four zero-current intervals in *i_a_* over one cycle. As IGBTs in the same phase sequence do not conduct simultaneously, no overlap occurs in fault effects, so the other two-phase currents remain distorted but do not reach zero.

Figure 5c shows the case where upper-bridge IGBTs in A-phase and B-phase experience OC faults. Segments I and III are impacted by individual IGBT faults, similar to a single IGBT OC fault waveform. Segment II remains unaffected, operating normally. In Segment IV, both *S*_1_ and *S*_3_ OC are in OC, resulting in zero intervals for both *i_a_* and *i_b_*. Since *i_c_* > 0, *D*_2_ remains at positive voltage and cannot conduct, meaning *D*_4_ and *D*_6_ do not turn off simultaneously, allowing at least two phases to conduct current. This fault waveform can be seen as an overlap of the individual *S*_1_ and *S*_3_ faults.

Figure 5d presents the scenario of a simultaneous OC fault in upper-bridge IGBT *S*_1_ of A-phase and lower-bridge IGBT *S*_6_ of B-phase. Segment I is fault-free, and Segment II involves only the *S*_6_ fault. In Segment III, *i_a_* ≤ 0 and with *S*_1_ in fault, A-phase current only flows through *D*_4_. Likewise, with *i_b_* ≥ 0 and *S*_6_ in fault, B-phase current can only pass through *D*_3_, preventing A- and B-phases from forming a current loop, meaning they do not conduct simultaneously during this stage. When *i_c_* < 0, *S*_5_ conducts while *S*_2_ is off, forming a current loop between B- and C-phases through *D*_3_ and *S*_5_, resulting in *i_a_* = 0 and *i_b_* = −*i_c_*. When *i_c_* ≥ 0, *S*_2_ conducts while *S*_5_ is off, forming a loop between A- and B-phases through *S*_2_ and *D*_4_, making *i_b_* = 0 and *i_a_* = −*i_c_*. Segment IV experiences only a single IGBT fault but with an increase in current amplitude across all phases.

**Figure 6:**
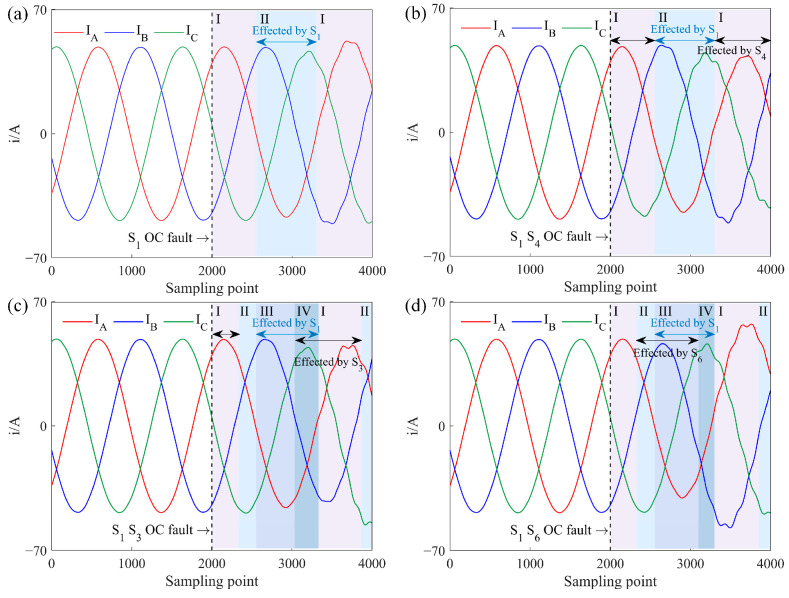
Three-phase current simulated waveform of IGBT OC fault under Task D, where I–IV represent different segments of the waveform. (**a**) *S*_1_ OC fault. (**b**) *S*_1_ *S*_4_ OC fault. (**c**) *S*_1_ *S*_3_ OC fault. (**d**) *S*_1_ *S*_6_ OC fault.

For Task D, where the motor is a PMSM operating in a rectifier state, a typical OC fault three-phase current simulated waveform is shown in Figure 6. Unlike the previous analysis, an OC fault does not cause segments of the current to reach zero. Instead, it leads to an imbalance in the three-phase power. This imbalance disrupts the symmetry of the current waveform, which can significantly affect the performance of the PMSM and lead to irregularities in power flow within the system.

The above content analyzed the four typical types of faults in the power converter under different operational tasks. Extending this analysis to all phases of IGBTs results in a total of 85 distinct fault types, as summarized in Table 3. Unique labels are assigned to each type of fault and used for neural network model training.

## 3. Approach

**Figure 7:**
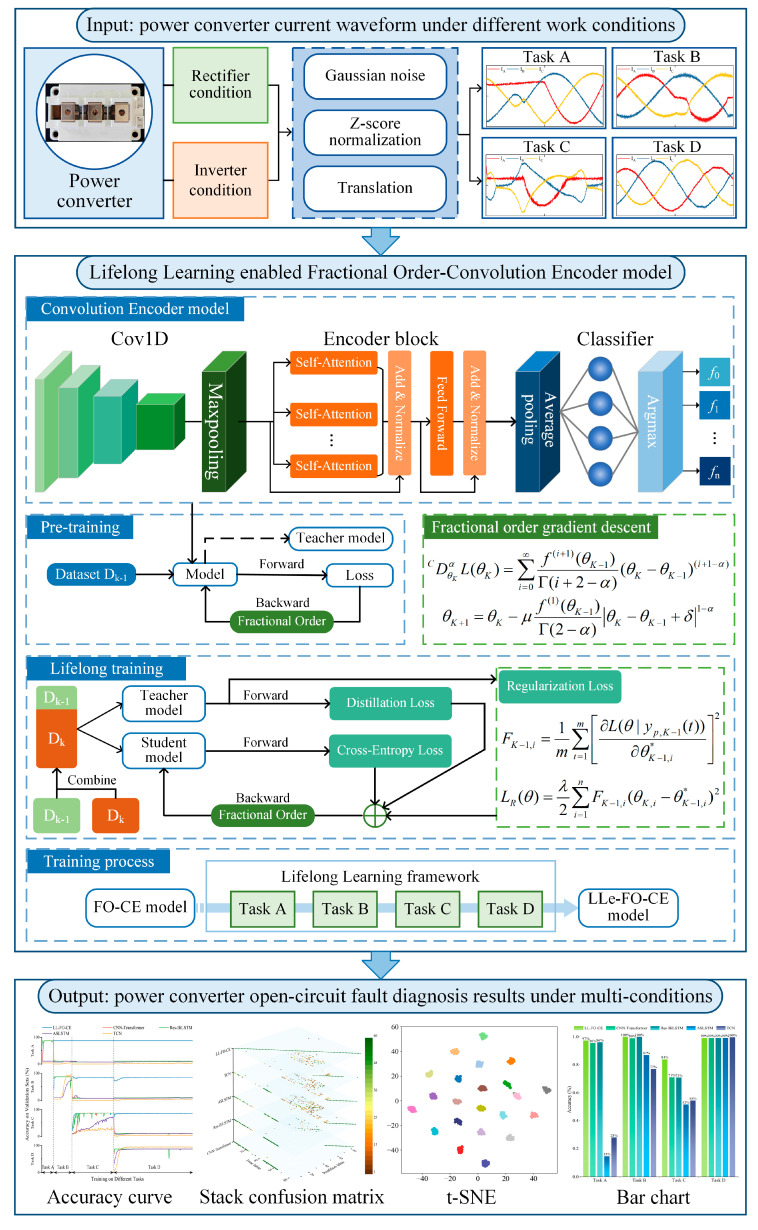
Lifelong learning-enabled fractional order-convolutional encoder model for open-circuit fault diagnosis of power converters under multi-conditions flow chat.

A lifelong learning-enabled fractional order-convolutional encoder (LL-FO-CE) model is proposed, with a flowchart shown in Figure 7. The proposed model is capable of continuously learning the three-phase current signals of a power converter across four different task types and accurately diagnosing faults in all previously learned tasks.

A simulation circuit is built on the MATLAB/Simulink platform, and a single cycle of current signals is collected while the power converter is operating stably, denoted as *I_original_*. To enhance the model’s generalization ability and robustness, data preprocessing is applied to the raw signals. Since OC faults in the power converter occur randomly and may appear at any point within a cycle, the original sample data are shifted along the time dimension to simulate current signals with faults occurring at different times. The data are cyclically shifted by a fixed step size, as shown in (1).

(1) $$\begin{array}{c} I_{1,shifted}=I_{original}[x_{t}],(t\geq 1,t\in N^{*})\\ I_{j,shifted}[x_{t}]=I_{j-1}[x_{t+p}],(j\geq 2,j\in N^{*}) \end{array}$$

The vector *I_j,shifted_* represents the *j*-th shifted sample, where *x_t_* is the sample point and *p* is the shifting step size. The shifted sample obtained is then used as the base sample for the next shift, with this process repeated until all data have been cycled through, ultimately yielding a total of *j* groups of fault samples. However, in practical applications, current signals are often affected by noise during acquisition. To simulate this interference, a random Gaussian white noise is applied to each sample group, as shown in (2).

(2) $$I_{j,noise}=I_{j,shifted}+c\cdot \frac{1}{\sqrt{2\pi }\sigma }\exp\left(-\frac{{\left(x-\mu \right)}^{2}}{2\sigma^{2}}\right)$$

where *μ* is the mean of the Gaussian distribution, *σ*^2^ is the variance, and *c* is the noise amplification factor. Setting *μ* = 0, *σ*^2^ = 1 and *c* = 10, we obtain the noise-augmented fault sample *I_j,noise_*. Given the large values in the original data, to prevent disproportionately large features from skewing the training results, the data are standardized using the Z-Score, as shown in (3).

(3) $$I_{j}=\frac{I_{j,noise}-\mu_{I}}{\sigma_{I}}$$

where *I_j_* represents the fault sample after data preprocessing, while *μ_I_* is the mean of *I_j,noise_*, and *σ_I_* is the standard deviation of *I_j,noise_*. Finally, by merging all the fault samples, we obtain the dataset *I* of three-phase current signals from the power converter under four different tasks, as shown in (4).

(4) $$I=\left[I_{1},I_{2},\ldots ,I_{j},\ldots ,I_{N}\right],(j=1,2,\ldots ,N)$$

The dataset *I* will be used for training and testing the LL-FO-CE model. The first part of the model is the convolutional module, which is designed to extract features from the data, as shown in (5) and (6) [39].

(5) $$H_{conv}=Conv1D(I)$$

(6) $$H_{pool}=Max(H_{conv})$$

where *H_conv_* is the output of the convolutional layer, and *H_pool_* is the output of the max-pooling layer. After the three-phase current signal samples are input into the model, they undergo multiple layers of 1D convolution and max-pooling to thoroughly extract the features of the fault samples. The hidden feature outputs from the convolutional module are then fed into the encoder module. First, the variables undergo positional encoding, as shown in (7)–(9) [40].

(7) $$X=H_{pool}+PE$$

(8) $$PE_{\left(pos,2i\right)}=\sin(pos/10000^{2i/d})$$

(9) $$PE_{\left(pos,2i+1\right)}=\cos(pos/10000^{2i/d})$$

where *pos* represents the position of each sample point in the sequence, *d* is the dimensionality of the position vector, which corresponds to the number of channels in the feature map output from the convolutional module, and *i* is the index of the dimension of the position vector. Then, the attention weights for the features are calculated using self-attention, as shown in (10) [41].

(10) $$Attention(Q,K,V)=\mathrm{Softmax}(\frac{Q\cdot K^{T}}{\sqrt{d_{k}}})V$$

√*d_k_* is the scaling factor, which helps prevent the vanishing gradients caused by excessively large products. *Q*, *K*, and *V* represent the Query, Key, and Value vectors in self-attention, as shown in (11)–(13).

(11) $$Q=X\cdot W_{Q}$$

(12) $$K=X\cdot W_{K}$$

(13) $$V=X\cdot W_{V}$$

*W_Q_*, *W_K_*, and *W_V_* are the weight parameter matrices, which are updated through model training. Then, by utilizing the multi-head attention mechanism, the model computes attention in parallel across different subspaces, as shown in (14).

(14) $$MultiHead(Q,K,V)=Concat(head_{1},head_{2},\ldots ,head_{h})W_{O}$$

The calculation formula for the *i*-th self-attention head is shown in (15).

(15) $$head_{i}=Attention(Q\times W_{Qi},K\times W_{Ki},V\times W_{Vi})$$

The output of each encoder layer will pass through a feedforward neural network consisting of two linear transformations and a nonlinear activation function, as shown in (16).

(16) $$FFN(x)=\max(0,xW_{1}+b_{1})W_{2}+b_{2}$$

*W* and *b* are the weight parameter matrix and bias parameter of the feedforward neural network, respectively, while *max* represents the activation function. The encoder module is composed of multiple identical encoders stacked together, with each layer including multi-head self-attention and a feedforward neural network. Thus, the output of a single-layer encoder can be expressed, as shown in (17) and (18).

(17) Z=LayerNorm(X+MultiHead(Q,K,V))

(18) $$Z^{\prime }=LayerNorm(Z+FFN(Z))$$

*Z* represents the output of the multi-head attention, *Z′* denotes the output of the encoder, and *LayerNorm* refers to layer normalization. The output of the encoder module is then passed through a fully connected layer, mapping it to the dimensionality of the number of classes, as shown in (19).

(19) $$H_{fc}=Z^{\prime }W_{fc}+b_{fc}$$

*H_fc_* represents the hidden state output from the fully connected layer, while *W_fc_* and *b_fc_* denote the weight parameter matrix and the bias parameter vector, respectively. Finally, the hidden state is transformed into a probability distribution using the *Softmax* function, and the final classification result is determined through *Argmax*, as shown in (20) and (21).

(20) $$P=Softmax(H_{fc})$$

(21) Class=Argmax(P)

The equations above outline the fundamental architecture and the forward propagation process of the proposed model. In the backpropagation and parameter update phase of the model, the fractional order derivative is utilized to optimize the gradient descent process [36]. The Caputo fractional order gradient descent is given by (22).

(22) DθαCL(θ)=∑i=I∞f(i)(θ0)Γ(i+1−α)(θ−θ0)(i−α)

*^C^D* is the Caputo fractional operator, *L*(*θ*) is the loss function, *α* is the order of the fractional derivative, and *I* is the smallest integer greater than *α*; thus, *I* − 1 < *α* < *I*. *θ* represents the neural network parameters, and *Γ* is the Gamma function. When *I* = 1 and 0 < *α* < 1, the expression is given by (23).

(23) DθKαCL(θK)=∑i=1∞f(i)(θK−1)Γ(i+1−α)(θK−θK−1)(i−α)

When *I* = 2, and 1 < *α* < 2, the expression becomes (24).

(24) DθKαCL(θK)=∑i=2∞f(i)(θK−1)Γ(i+1−α)(θK−θK−1)(i−α)

Therefore, the expression for the fractional order when 0 < *α* < 2 is given by (25).

(25) DθKαCL(θK)=∑i=0∞f(i+1)(θK−1)Γ(i+2−α)(θK−θK−1)(i+1−α)

By applying the Taylor series expansion to (25) up to the first term, we obtain the fractional order loss function for the case when 0 < *α* < 2, as shown in (26).

(26) DθKαCL(θK)=f(1)(θK−1)Γ(2−α)θK−θK−1+δ1−α

*δ* is a small positive number introduced to prevent division by zero. Therefore, the parameter update expression based on the fractional order gradient descent is shown in (27).

(27) $$\theta_{K+1}=\theta_{K}-\mu \frac{f^{\left(1\right)}(\theta_{K-1})}{\Gamma (2-\alpha )}{\left|\theta_{K}-\theta_{K-1}+\delta \right|}^{1-\alpha }$$

This method allows the neural network to update parameters using fractional order gradients during backpropagation, enabling the model to quickly find the global optimal solution and avoid getting trapped in local optima.

The above process allows the model to train optimally for fault diagnosis tasks in a single state of the power converter. However, when the state changes, the model will be unable to recognize new fault features and will need to be retrained with new fault samples. To enable the model to continue learning new fault samples, a multilevel lifelong learning framework has been designed. This framework includes randomly inserting samples from previous tasks into the new batch of training data, learning soft labels from previous tasks, and adding a regularization penalty term to the loss function.

First, a replay buffer containing randomly selected fault samples from previous tasks is constructed, as shown in (28) [42].

(28) $$D_{A,re}=[I_{j}],I_{j}\subseteq D_{A},j\sim U(1,m)$$

*I_j_* represents the fault samples from Task A, where *j* is a random number in the interval (1, *m*), and *m* is the total number of samples in Task A. This operation is performed at the beginning of each iteration cycle when the model is learning a new task, to update the samples in the replay buffer. The samples from *D_A,re_* are combined with the current batch of training data *D_B_*, as shown in (29).

(29) $$D_{AB}=D_{A,re}+D_{B}$$

*D_AB_* contains samples from both the previous Task A and the current Task B, which are used for model training. To maintain consistency across the two different tasks, the model learns the soft labels from the previous task, as shown in (30) [43].

(30) $$L_{KD}(\theta )=(1-\beta )L_{CE}(\theta |y_{s}(x_{AB}))+\beta \cdot L_{KL}\left\{\theta |\sigma \left[\frac{y_{s}(x_{AB})}{T}\right],\sigma \left[\frac{y_{T}(x_{AB})}{T}\right]\right\}$$

*L_CE_* is the cross-entropy loss function, while *L_KL_* denotes the Kullback–Leibler (KL) divergence loss function. The *σ* represents the *Softmax* function, and *y_T_* and *y_S_* refer to the model’s predictions on the previous task and the current task, respectively. *β* is a weight parameter, and *T* is the temperature coefficient. The loss *L_KD_* consists of two parts: one is the hard target loss *L_CE_*, which measures the discrepancy between the model’s predictions on the current task and the true labels. The other part is the soft target loss *L_KL_*, which indicates the difference between the model’s output on the previous task and the soft labels. By adjusting the size of *β*, the model’s focus during the learning of new tasks can be controlled. A larger *β* places more emphasis on retaining previous knowledge, while a smaller *β* favors the learning of the new task. The temperature coefficient *T* is used to regulate the difficulty of learning the knowledge from previous tasks; a larger *T* facilitates the acquisition of knowledge from earlier tasks.

To mitigate the model’s forgetting speed regarding previous tasks, a regularization penalty term that measures the importance of the model parameters is added to the loss function. For Task D, the optimal parameters *θ* should maximize the conditional probability *P*(*θ*|*D*), which can be expressed using Bayes’ theorem as (31) [44].

(31) $$\log P(\theta |D)=\log\frac{P(D_{B}|\theta D_{A})P(\theta D_{A})}{P(D_{A}D_{B})}$$

*D_A_* and *D_B_* are two distinct tasks that make up Task D. Since *D_A_* and *D_B_* are independent of each other, we obtain (32).

(32) $$\log P(\theta |D)=\log P(D_{B}|\theta )+\log P(\theta |D_{A})-\log P(D_{B})$$

Under the given parameters, log*P*(*D_B_*|*θ*) represents the loss function for Task *D_B_*, denoted as −*L_B_*(*θ*), and log*P*(*D_B_*) is a constant. Thus, the optimization objective is expressed as (33).

(33) $$\max\log P(\theta |D)=\max(-L_{B}(\theta )+\log P(\theta |D_{A}))$$

Simplifying the right side of the equation yields (34).

(34) $$\max\log P(\theta |D)=\min(L_{B}(\theta )-\log P(\theta |D_{A}))$$

For the posterior probability *P*(*θ*|*D_A_*), it can be approximated as a Gaussian distribution that conforms to the prior probability *P*(*D_A_*|*θ*). Let *f*(*θ*) = log *P*(*D_A_*|*θ*), and expand it up to the third term at the optimal parameter θ^*^_A_ using the Taylor formula, as shown in (35).

(35) $$f(\theta )=f(\theta_{A}^{*})+f^{\prime }(\theta_{A}^{*})(\theta -\theta_{A}^{*})+\frac{1}{2}f^{\prime \prime }(\theta_{A}^{*}){\left(\theta -\theta_{A}^{*}\right)}^{2}+o(\theta_{A}^{*})$$

Since θ^*^_A_ is the optimal solution for *f*(*θ*), it follows that *f′*(θ^*^_A_) = 0. Substituting the probability density function of *f*(*θ*) gives us (36).

(36) $$f(\theta )=\log\frac{1}{\sqrt{2\pi }\delta }-\frac{{\left(\theta -\mu \right)}^{2}}{2\delta^{2}}\approx f(\theta_{A}^{*})+\frac{1}{2}f^{\prime \prime }(\theta_{A}^{*}){\left(\theta -\theta_{A}^{*}\right)}^{2}$$

By solving, we obtain *μ* = θ^*^_A_ and *δ*^2^ = −1/*f″*(θ^*^_A_). Substituting these into (34) gives us the expression shown in (37).

(37) $$\max\log P(\theta |D)=\min(L_{B}(\theta )-\log(1/\sqrt{2\pi }\delta )-\frac{1}{2}f^{\prime \prime }(\theta_{A}^{*}){\left(\theta -\theta_{A}^{*}\right)}^{2})$$

log [1/(√2*π*)*δ*] is a constant. Based on the optimization objective, we can construct the loss function *L_REG_* as shown in (38).

(38) $$L_{REG}(\theta )=L_{B}(\theta )-\frac{1}{2}f^{\prime \prime }(\theta_{A}^{*}){\left(\theta -\theta_{A}^{*}\right)}^{2}$$

where *f″*(θ^*^_A_) can be represented by the diagonal elements of the Fisher information matrix −*F_A,i_*, as shown in (39) [45].

(39) $$F_{A,i}=\frac{1}{m}{\sum_{t=1}^{m}\left[\frac{\partial L(\theta |y_{p,A}(t))}{\partial \theta_{A,i}^{*}}\right]}^{2}$$

θ^*^_A_ represents the network parameters of the neural network on Task *D_A_*, *y_p,A_*(*t*) is the predicted value on *D_A_*, and *m* is the total amount of data in *D_A_*. By calculating the partial derivative of the loss function of all predicted values with respect to the network parameters θ^*^_A_, the importance of weight *F_A,i_* of the parameters for Task *D_A_* can be obtained. The neural network will continue training on Task *D_B_*, and the loss function will be constructed by incorporating *F_A,i_* as shown in (40).

(40) $$L_{REG}(\theta_{B,K+1})=L_{CE}(\theta_{B,K})-\frac{\lambda }{2}\sum_{i=1}^{n}F_{A,i}{\left(\theta_{B,i}-\theta_{A,i}^{*}\right)}^{2}$$

The loss function *L_KD_* is combined with *L_REG_*, and the parameters of the LL-FO-CE model are updated through fractional-order gradient descent during backpropagation, resulting in the parameter update expression as shown in (41).

(41) θN,K+1=θN,K−μDθN,KαCLKD(θN,K)+μ∂LREG(θN,K)∂θ=θN,K−μ1Γ(2−α)[∂LCE(θN,K−1)∂θ−β∂LCE(θN,K−1)∂θ+βLKL(θN,K−1)∂θ]θK−θK−1+δ1−α+μ∂LCE(θN,K−1)∂θ−μλm∑i=1n∑x=1m∂LCE(θ|yN−1(x))∂θN−1,i*2(θN,i−θN−1,i*)∂θN,i∂θ

## 4. Simulation

In this section, the proposed LL-FO-CE model will be verified in detail for its diagnostic performance of power converter OC faults under different tasks. Additionally, LL-FO-CE will be compared with four state-of-the-art (SOTA) models that have been validated for their accuracy in diagnosing OC faults in power converters: CNN-Transformer [46], Res-BiLSTM [47], ASLSTM [35], and TCN [48]. These models are innovative and demonstrate exceptional performance in OC fault diagnosis, as reported in their original publications.

Co-simulation experiments were conducted using Python and MATLAB/Simulink, specifically with Python version 3.9 and MATLAB/Simulink version R2022b. Python is an open source programming language developed and maintained by a global community of developers. A deep neural network is constructed using the PyTorch 2.3.0 framework, with CUDA employed to accelerate the training process. Simulation circuits are developed in MATLAB/Simulink R2022b to generate fault simulation data for training and testing the neural network model.

### 4.1. Fractional Order Design

According to Section 3, the fractional order significantly influences the speed of parameter updating and convergence in the neural network model. The order also determines the memory length and strength of the fractional order derivatives. An appropriate order can more effectively capture the long-term dependencies within the data, thereby enhancing the model’s diagnosis performance. However, the optimal order often varies across different types of tasks, necessitating exploration to identify the most suitable order for the model.

**Figure 8:**
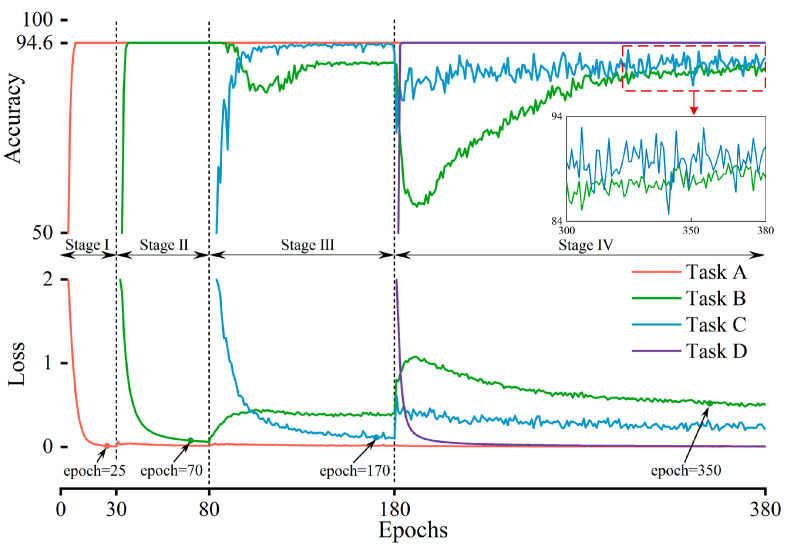
The optimal training epochs for the model across different tasks.

It is first necessary to determine the optimal number of training epochs for the model on different tasks. Let LL-FO-CE be continuously trained on four tasks, and the loss curves and validation set accuracy curves during training are shown in Figure 8. The upper half of the figure displays the model’s accuracy on the validation set, while the lower half shows the training loss. Curves of different colors represent the model’s accuracy or loss for each task. The stages indicate different training phases: in Stage I, the model is trained on Task A; in Stage II, it switches to Task B and continues training, and so on. In Stage I, when the epoch reaches 25, the accuracy converges to its maximum value, and the loss converges to its minimum. As the training stages progress, the optimal number of iterations required for the model to converge on each task gradually increases. This is because the model requires more training epochs to solidify its memory of the previous tasks. Upon discussion, the optimal number of iterations for the model in Stage II, Stage III, and Stage IV are found to be 40, 90, and 170, respectively.

**Figure 9:**
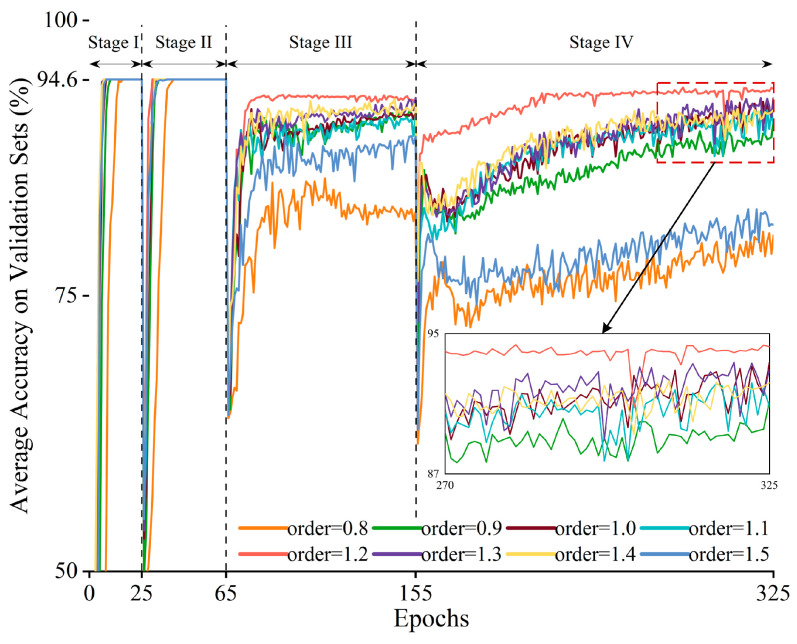
Optimal fractional order of derivative.

The optimal training epochs above represent the best values for neural network models using traditional integer-order gradient descent. Building on this, we further investigate the optimal order for the model under fractional order gradient descent. Figure 9 shows the average accuracy curve on validation sets of previously learned tasks under different fractional orders, where order = 1 represents traditional integer-order gradient descent. During the first two stages, all orders converge to the highest accuracy. In Stage III, differences in convergence speed and maximum accuracy emerge across orders. In Stage IV, integer-order gradient descent requires 325 epochs to converge, with a maximum average accuracy of 92%. In contrast, fractional order gradient descent with an order of 1.2 converges by epoch 210, achieving a maximum average accuracy of 94% across all validation sets, a 2% improvement over integer-order descent. This indicates that with order = 1.2, fractional order gradient descent enables faster convergence and higher accuracy.

Combined with the above studies, the optimal hyperparameters for the training process of LL-FO-CE are shown in Table 4. The fundamental training parameters, such as epoch, learning rate, and batch size, are also applicable to the other SOTA models discussed in the paper.

**Figure 10:**
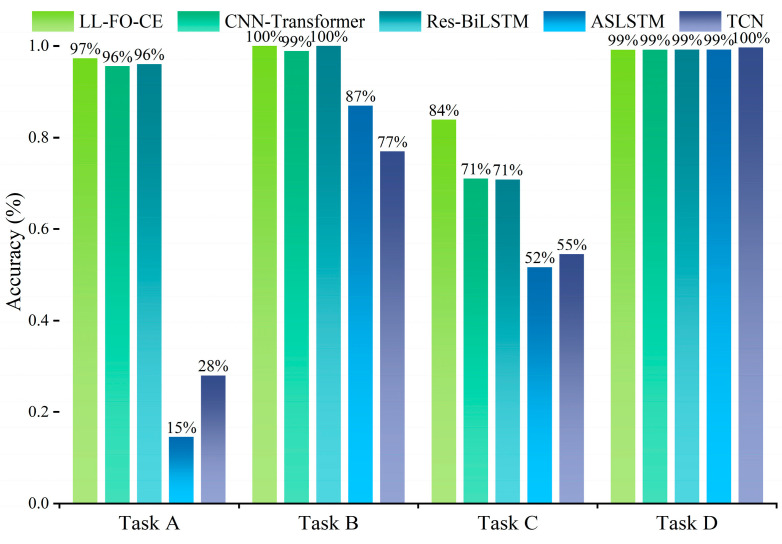
The fault diagnosis accuracy of each model on the random pulse noise signal dataset.

Since fractional order gradient descent allows the model to find a globally optimal solution to the task, this means that the model still performs well when the task becomes more complex. The following discussion is carried out to verify this idea. Let LL-FO-CE and other SOTA models be trained separately on four tasks and subsequently tested on a dataset with random pulse noise signals applied. The accuracy results are presented in Figure 10. LL-FO-CE achieves the highest classification accuracy on the test set for each task, with an average accuracy of 95%. In contrast, the other four SOTA models exhibit lower average accuracies of 91.25%, 91.5%, 63.25%, and 65%, respectively. This demonstrates that fractional order enhances the model’s robustness to noisy data.

**Figure 11:**
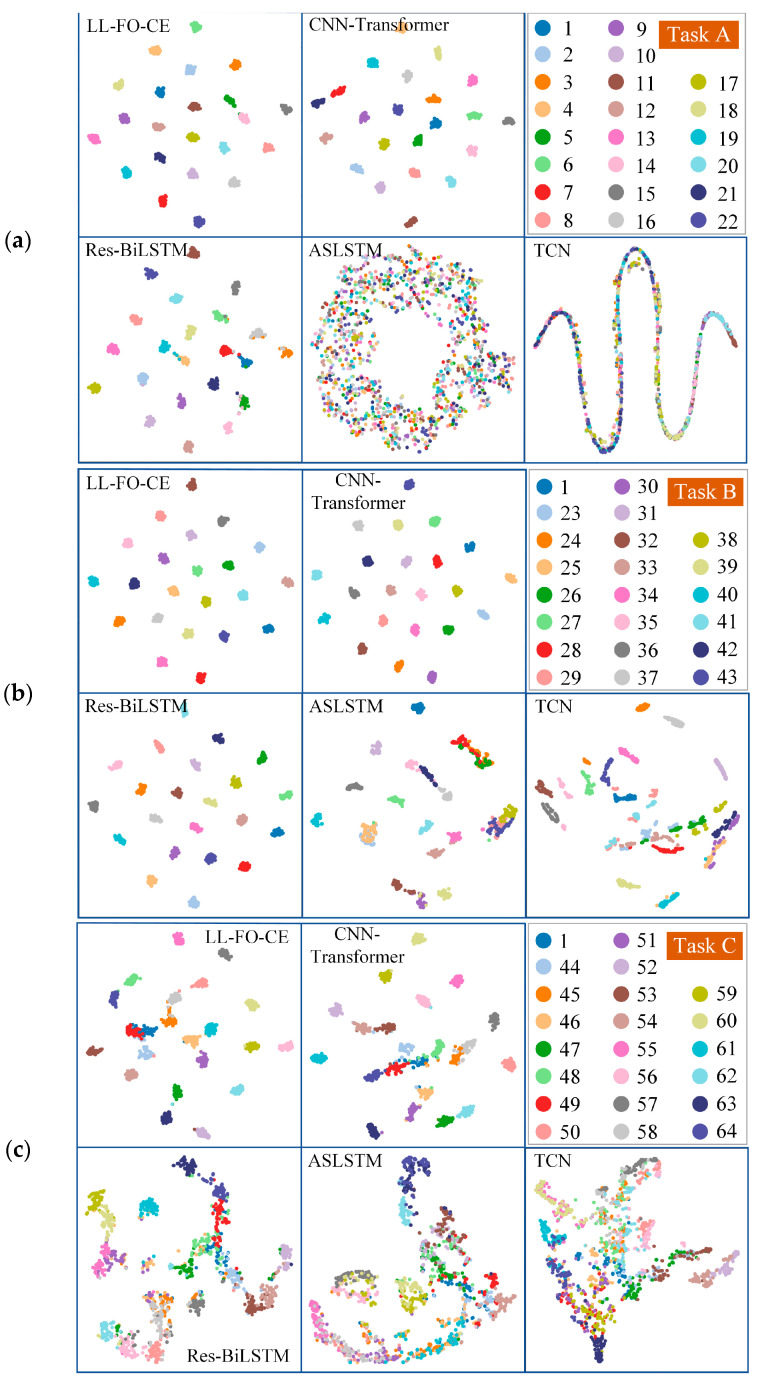

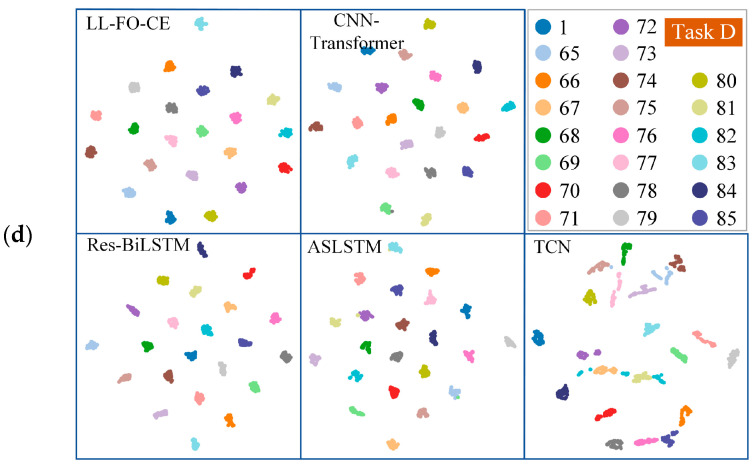
The t-SNE plots of each model on the random pulse noise signal dataset. (**a**) The t-SNE plot for each model on Task A. (**b**) The t-SNE plot for each model on Task B. (**c**) The t-SNE plot for each model on Task C. (**d**) The t-SNE plot for each model on Task D.

To further investigate the diagnostic performance of each model on the random pulse noise signal test set, the high-dimensional data output from the model’s hidden layer is downscaled and visualized using t-distributed stochastic neighbor embedding (t-SNE), as shown in Figure 11. Each subgraph corresponds to a specific task, and the points of different colors represent different categories of fault samples; their correspondences are indicated in the upper right corner of each subgraph. The distribution of color blocks for each category in the picture can reflect the diagnostic performance of the model on the corresponding fault samples of the category. If the data points of the same category are closely clustered together, with a clear separation between points of different categories, it indicates that the model can accurately identify and classify different types of samples, signifying high fault diagnosis accuracy. Figure 11a shows the t-SNE plots for each model in Task A. The LL-FO-CE achieves clear categorization of the data, exhibiting tight clustering within each category and significant separation between different categories. This suggests that the model effectively recognizes the features of various sample categories, leading to accurate diagnoses. The clustering results of the CNN-Transformer indicate that the distance between certain categories is smaller, and the boundaries are more ambiguous. This suggests that the model’s ability to differentiate the features of various categories is somewhat inadequate. The Res-BiLSTM can form clusters for samples in most categories; however, the tightness of the data within each category is relatively low, and there is some category overlap. Specifically, data from different categories are clustered in the same region, indicating that the model misdiagnoses some fault samples, which results in lower diagnostic accuracy. The ASLSTM combines a significant number of data points from different categories, resulting in indistinct category boundaries. This suggests that its diagnostic performance on the random pulse noise signal test set is poor, and its robustness is weak. The TCN distributes all data points along a continuous curve, indicating that it recognizes temporal relationships in the data but fails to identify other features, resulting in no obvious clustering structure. Overall, the LL-FO-CE demonstrates optimal performance across all tasks, exhibiting clear category boundaries and tight clustering. This indicates that the model can maintain strong diagnostic performance on the random pulse noise signal test set, further proving its robust anti-interference capability.

### 4.2. Performance Study of the Lifelong Learning Framework

**Figure 12:**
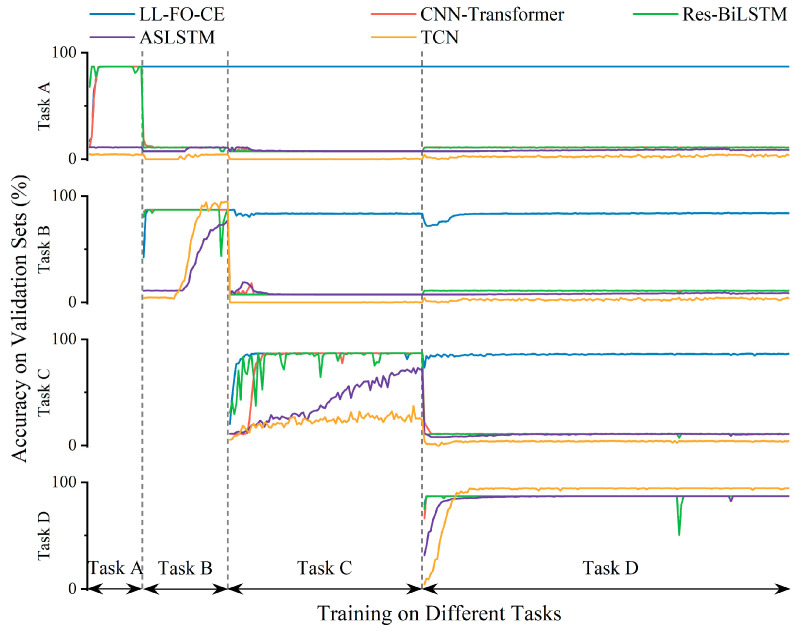
Accuracy of each model on the validation set during continuous learning of different tasks.

To verify the lifelong learning capability of the proposed model, the following experiments were conducted in this study. The models were trained sequentially from Task A to Task D, and the accuracy of each model on the validation set for all learned tasks was evaluated at the end of each epoch, as shown in Figure 12. In Stage I, while training on Task A, all models except ASLSTM and TCN achieved the highest accuracy. In Stage II, when the models switched to Task B, all of them attained high accuracy on the validation set for Task B before the end of the training phase. However, for the validation set of Task A, the accuracy of CNN-Transformer and Res-BiLSTM dropped below 10% within just a few epochs, while only the LL-FO-CE maintained high accuracy on Task A. In Stage III, all models except LL-FO-CE forgot the knowledge of Task B and could only learn and remember features from Task C. In the final stage, all models achieved high accuracy on Task D, but only LL-FO-CE was able to maintain high accuracy across the other three tasks simultaneously. This indicates that neural network models typically struggle to retain memory for single tasks, and once they engage in continuous learning of different tasks, they tend to forget previously acquired knowledge. In contrast, the lifelong learning framework proposed in this paper enables the LL-FO-CE to maintain long-term memory of all learned knowledge, ensuring that it retains high accuracy on previous tasks and avoids the catastrophic forgetting that often occurs when learning different tasks.

### 4.3. Research on Fault Diagnosis Performance

**Figure 13:**
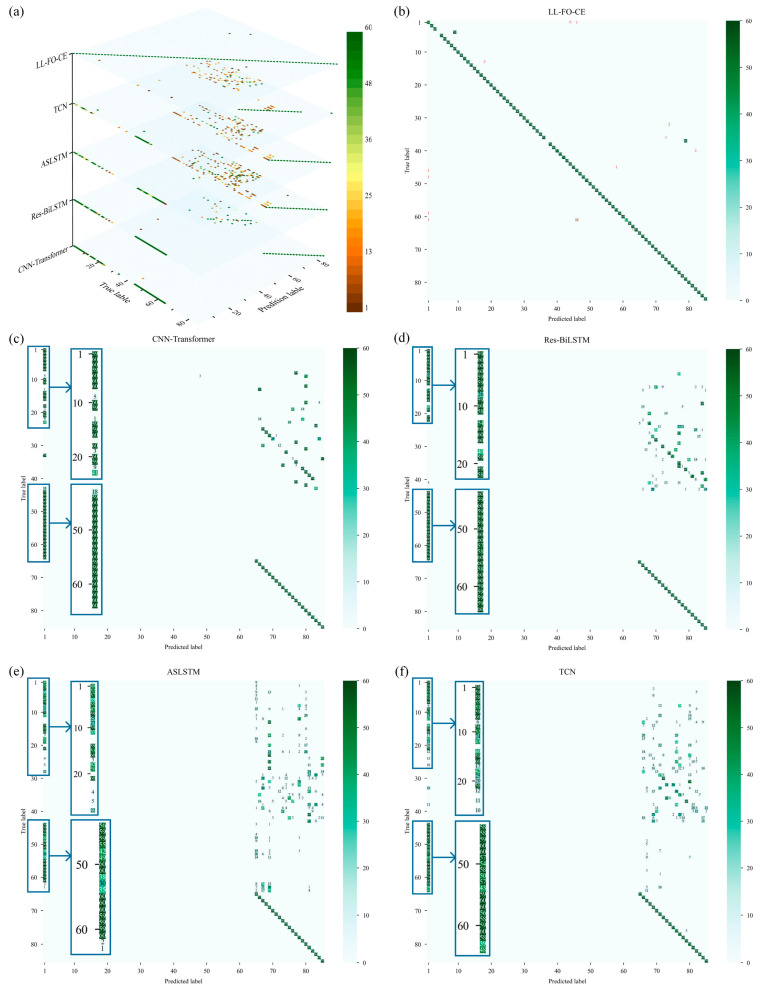
Stacked plots of diagnosis results and confusion matrices for all fault categories of each model. (**a**) Stacked confusion matrix of fault diagnosis for each model on the test set. (**b**) Confusion matrix of fault diagnosis for LL-FO-CE model on the test set. (**c**) Confusion matrix of fault diagnosis for CNN-Transformer model on the test set. (**d**) Confusion matrix of fault diagnosis for Res-BiLSTM model on the test set. (**e**) Confusion matrix of fault diagnosis for ASLSTM model on the test set. (**f**) Confusion matrix of fault diagnosis for TCN model on the test set.

A fault sample test set containing all 85 fault categories was constructed to validate the fault diagnosis performance. The LL-FO-CE model was trained continuously on four tasks alongside other SOTA models, and the diagnosis results for all fault categories were validated on the test set. The stacked plots of the diagnosis results and confusion matrix for each model are shown in Figure 13.

Figure 13b–f shows the confusion matrices of the diagnosis results of each model in the validation set, where the X-axis represents the true labels of the samples and the Y-axis represents the predicted labels of the models. Figure 13a shows a stacked plot of the confusion matrices, where the Z-axis of the plot represents the different models. It was shown that the diagnosis results of the other SOTA models on the first 64 category samples significantly deviated from the true categories and failed to accurately classify the faulty samples. These samples all belonged to Task A, Task B, and Task C, further suggesting that these SOTA models experienced catastrophic forgetting when learning different tasks consecutively, resulting in a significant decrease in diagnosis ability on previous tasks. In addition, these models judged the vast majority of fault samples with a predictive label of 1 as “no fault”, which shows that they lost the ability to recognize faults in the first 64 categories. For all 85 fault categories, the diagnosis average accuracies of CNN-Transformer, Res-BiLSTM, ASLSTM, and TCN are 25.83%, 25.87%, 25.85%, and 25.88%, respectively.

The diagnosis of fault samples by LL-FO-CE primarily focuses on the main diagonal of the confusion matrix, achieving an accuracy of 96.89%. This indicates that the proposed model exhibits accurate classification ability on all 85 fault categories. Although the model deviates from the true label on the classification of a few samples, almost all samples successfully identify the faults, i.e., the predicted labels are not equal to 1. The experimental results validate that LL-FO-CE is able to accurately diagnose OC faults of IGBTs of the power converter under multiple operating conditions and demonstrates high diagnosis accuracy.

**Figure 14:**
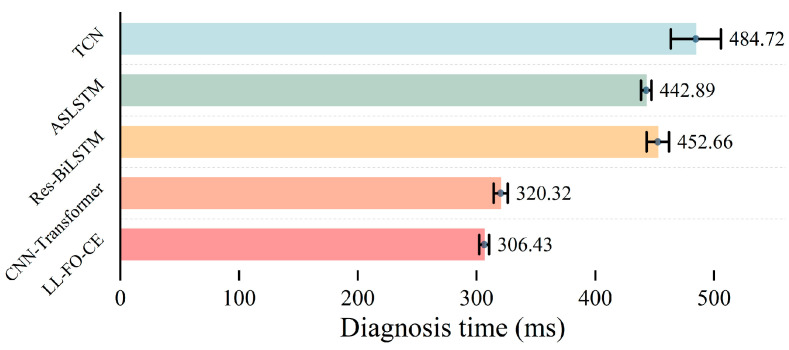
Comparison of diagnosis time of each model in the test set.

Figure 14 illustrates the average diagnosis time and its standard deviation for the five models evaluated on the test set. The left axis represents the different model categories, while the bottom axis indicates the diagnosis time, defined as the computation time of each model. The rightmost side of each bar is labeled with the specific time value with the error bar indicating the standard deviation. As illustrated in the figure, LL-FO-CE exhibits the shortest diagnostic time, with an average of 306.43 ms and the smallest standard deviation of 4.2 ms. This indicates that its computation time is stable, minimally affected by input samples, and demonstrates strong robustness. In comparison, the average computation time and standard deviation for the CNN-Transformer are higher, at 320.32 ms and 6 ms, respectively. The average computation time for Res-BiLSTM is 452.66 ms, which is 47.7% higher than that of LL-FO-CE. Additionally, its standard deviation is relatively large at 9.5 ms, suggesting that the model is unstable. The average diagnostic time for ASLSTM is 442.89 ms, and the standard deviation is 4.44 ms. The average computation time and standard deviation of TCN are 484.72 ms and 21.13 ms, respectively, which are the maximum values among the five models, indicating that the model has not only high computational complexity but also poor stability. Overall, LL-FO-CE demonstrates the highest computational efficiency among the five models and outperforms the others in terms of stability, with high real-time performance and robustness, and is suitable for real-time fault diagnosis tasks.

## 5. Semi-Physical Experiments

**Figure 15:**
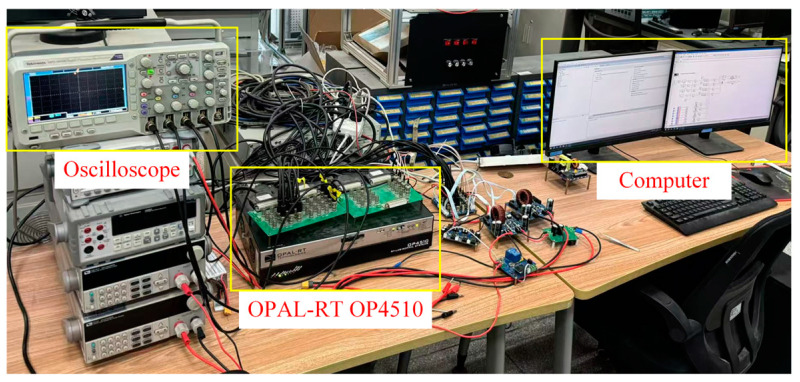
Semi-physical virtual simulation system for power converter fault diagnosis.

To further verify the reliability of the proposed fault diagnosis method, a semi-physical virtual simulation system for power converter fault diagnosis has been established using the OPAL-RT OP4510 real-time simulator, a Tektronix (Beaverton, OR, USA) oscilloscope, and a desktop computer equipped with an AMD R5-7500F CPU, an RTX 4060 Ti GPU, and 32 GB of RAM. The CPU is manufactured by Advanced Micro Devices, Inc. (Santa Clara, CA, USA), while the GPU is produced by NVIDIA Corporation (Santa Clara, CA, USA). The experimental setup is shown in Figure 15. The OPAL-RT OP4510 is used to generate the control signals and run the simulation model, which operates on RT-LAB, the industrial-grade real-time simulation system developed by OPAL-RT (Montreal, QC, Canada). The oscilloscope monitors and collects the current signals generated by the OP4510 in real time, producing waveform files. The desktop computer is used to run the fault diagnosis program.

The workflow of the semi-physical virtual simulation experiment system is as follows: First, build and compile the simulation model file according to the RT-LAB standard on the computer. Next, transfer the compiled file to the OP4510 real-time simulation platform. After that, start the simulation model on the OP4510 and perform the corresponding control operations on the model. At the same time, an oscilloscope is used to monitor and collect the current signal, generating the corresponding current waveform file. During this process, the computer will run the fault diagnosis program synchronously to analyze the waveform data generated by the oscilloscope in depth, so as to realize the accurate detection of faults and the precise location of the failed device.

The real-time emulator OP4510 generates and controls PWM signals to drive a power converter emulation circuit. In this circuit, each IGBT device corresponds to a specific PWM signal. When all PWM signals are functioning normally, the power converter maintains a stable operating state. However, if a PWM signal is disconnected, the corresponding IGBT device will enter a shutdown state, thereby simulating an OC fault scenario of the power converter in real applications. The system adopts the double closed-loop control method based on the *d-q* rotating coordinate system, which is mainstream in the engineering field. The main parameter settings are detailed in Table 5 to ensure the accuracy and stability of the system control.

**Figure 16:**
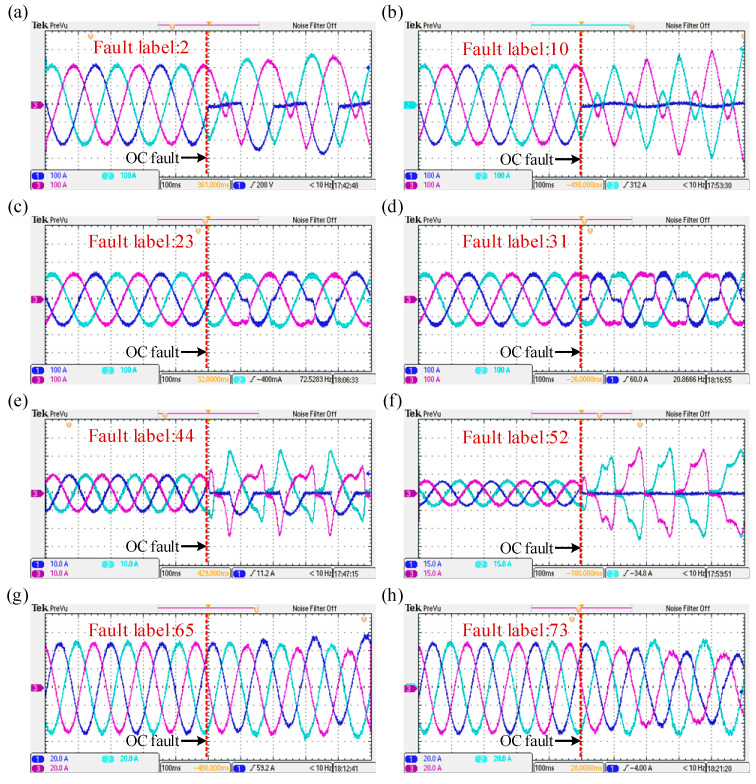
Partial fault waveforms for semi-physical virtual simulation system. (**a**) Fault waveform of a three-phase circuit with fault label 2. (**b**) Fault waveform of a three-phase circuit with fault label 10. (**c**) Fault waveform of a three-phase circuit with fault label 23. (**d**) Fault waveform of a three-phase circuit with fault label 31. (**e**) Fault waveform of a three-phase circuit with fault label 44. (**f**) Fault waveform of a three-phase circuit with fault label 52. (**g**) Fault waveform of a three-phase circuit with fault label 65. (**h**) Fault waveform of a three-phase circuit with fault label 73.

A validation set of three-phase current data, encompassing 85 fault categories, was obtained through the semi-physical virtual simulation system. Each fault category includes 60 sets of three-phase current signal samples, with eight typical fault waveforms illustrated in Figure 16. In comparison to the Simulink simulation waveforms, these waveforms exhibit noticeable burrs and noise interference, which may increase the difficulty of fault diagnosis.

Four evaluation metrics are used to assess the effectiveness of fault diagnosis: accuracy, precision, recall, and F1 score. Accuracy represents the number of correctly diagnosed samples as a proportion of the total number of samples, reflecting the overall effectiveness of the fault diagnosis results, as shown in (42).

(42) $$Accuracy=\frac{TP+TN}{N}$$

where *TP* is the number of samples correctly diagnosed as positive classes, which means that no faulty samples were correctly diagnosed. *TN* is the number of samples correctly diagnosed as negative classes, which means that faulty samples were correctly diagnosed.

The precision indicates the proportion of samples predicted by the model to belong to each category that actually do belong to that category. It is used to measure the accuracy of the model’s predictions for each category. The global precision is calculated by Macro-Average as shown in (43).

(43) $$MacroPrecision=\frac{1}{N}\sum_{i=1}^{N}\frac{TP_{i}}{TP_{i}+FP_{i}}$$

where *TP_i_* denotes the number of samples correctly predicted by the model to be in category *i*, *FP_i_* denotes the number of samples incorrectly predicted by the model to be in category *i*, and *N* represents the total number of samples across all categories. Macro-Averaging is applicable when the number of samples is equal for all categories and their importance is considered uniform.

Recall indicates, for each category, the proportion of samples that correctly belong to that category and are correctly predicted by the model as being in that category. It is used to measure the model’s ability to recognize each category. The global recall is calculated by Macro-Average as in (44).

(44) $$MacroRecall=\frac{1}{N}\sum_{i=1}^{N}\frac{TP_{i}}{TP_{i}+FN_{i}}$$

F1 Score is the harmonic mean of precision and recall, providing a more comprehensive assessment of the model’s performance metrics in terms of precision and recall. The global F1 Score is calculated by Macro-Average as in (45).

(45) $$MacroF1score=\frac{1}{N}\sum_{i=1}^{N}\frac{2*\frac{TP_{i}}{TP_{i}\;+\;FP_{i}}*\frac{TP_{i}}{TP_{i}\;+\;FN_{i}}}{\frac{TP_{i}}{TP_{i}\;+\;FP_{i}}+\frac{TP_{i}}{TP_{i}\;+\;FN_{i}}}=\frac{2}{N}\sum_{i=1}^{N}\frac{TP_{i}}{2TP_{i}+FN_{i}+FP_{i}}$$

**Figure 17:**
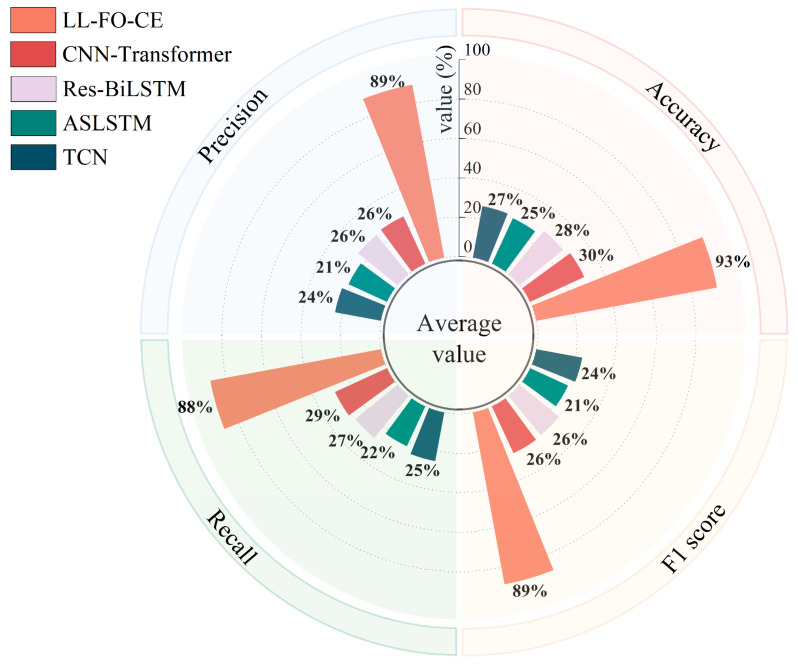
Overall average of various models across 10 predictions under all tasks.

The models were permitted to make ten predictions on the validation set, and their fault diagnosis performance was evaluated using the aforementioned metrics. The results are presented in Figure 17, and Table 6 displays the average of accuracy, precision, recall, and F1 score for each model based on ten predictions across various tasks.

For Task A, Task B, and Task C, the accuracy of LL-FO-CE is 92.9%, 94.5%, and 87.9%, respectively, which shows the best performance among all models. The precision, recall, and F1 scores also represent the highest values across all models, indicating strong diagnosis accuracy and fault identification capability. In contrast, the other models struggled with fault recognition and diagnosis on the validation set for the first three tasks. This limitation arose because they could not maintain long-term memory of different tasks and gradually forgot the knowledge of previous tasks while continuously learning new tasks.

For Task D, the accuracy of LL-FO-CE, CNN-Transformer, Res-BiLSTM, ASLSTM, and TCN are 98.5%, 98%, 97.1%, 94.3%, and 92.9%, respectively. The precision scores are 98.6%, 98.1%, 98%, 92.1%, and 92.3%, respectively. The recall scores are 98.5%, 98%, 97.1%, 90.8%, and 92.4%. The F1 scores are 98.5%, 98.1%, 97.6%, 90.8%, and 92.2%, respectively. All models demonstrate improved performance on this task because Task D is the final task to be learned. However, the presence of disturbances in the fault sample waveforms results in a certain degree of degradation in the diagnosis performance of the other models. LL-FO-CE enhances the backpropagation optimization process through fractional order derivatives, which are highly robust, thereby maintaining superior diagnosis performance compared to other SOTA models when applied to actual fault waveforms with disturbances.

Figure 17 illustrates the overall average of 10 predictions made by each model across all tasks in the validation set. The different colored areas represent various metrics, while distinct colored bars indicate different models. For all 85 fault categories, LL-FO-CE demonstrates optimal performance across all metrics, achieving an average diagnosis accuracy of 93%. The other models, which can only perform better on Task D, exhibit performance averages ranging from 20% to 30% across all tasks. This figure visualizes the performance disparity between the proposed model and other SOTA models in the validation set, highlighting the advantages of the proposed model in diagnosing power converter faults under varying conditions.

## 6. Conclusions

A lifelong learning-enabled fractional order-convolutional encoder model is proposed for continuous learning as well as accurate diagnosis of OC faults in power converters under multi-conditions. The conclusions are as follows:

1. A convolutional encoder model is proposed for learning and diagnosing OC faults in the power converter. The model automatically extracts time series features from three-phase current fault signals and analyzes the relative positional relationships between each signal using a convolution module. It employs an encoder module to identify and classify these features, thereby enabling the automatic learning and diagnosis of fault samples.

2. The optimization process of the neural network model is improved by using the global search and smooth gradient properties of fractional order. The global search property enables the model to comprehensively consider historical gradient information during backpropagation, effectively exploits the long-term dependencies of fault samples. The smooth gradient property can effectively reduce the oscillation of the optimization path and accelerate the speed of the model convergence to the global optimal solution. Compared to integer-order models, the diagnosis accuracy improves by 2%, and the number of training epochs decreases by 115. It shows stronger robustness to abnormal noise signal samples, with an accuracy improvement of 2.75% compared to other models.

3. The designed multilevel lifelong learning framework equips the model with the ability to continuously learn various tasks. By incorporating loss function penalty terms, random replay, and soft labeling for previous tasks, the model is able to continue to learn new fault samples after training is completed and maintains stability and consistency across all fault classes. The study shows that the proposed model achieves 96.89% on the test set and 93% on the validation set after continuously learning fault samples from four different tasks of the power converter, which solves the problem of decreasing diagnosis accuracy of the conventional models when the operating state of the power converter changes, and is of great engineering practical significance to improve the operation safety and reliability of the motor drive system.

4. The lifelong learning-enabled fractional order-convolution encoder model proposed in this study has achieved significant results in diagnosing OC faults of power converters under multi-conditions. This approach has a wide range of applications in intelligent operation and maintenance systems for electric vehicles, renewable energy generation, rail transportation, etc. By facilitating real-time monitoring and early diagnosis of faults, it enables early warning and localization of faults, thus improving system safety and reliability while reducing maintenance costs. Future research will further focus on life prediction and reliability management technologies for power semiconductor devices based on Prognostics and Health Management. This will provide health monitoring and intelligent operation and maintenance support throughout the entire life cycle of power electronic systems.

## Figures and Tables

**Table 1:** Four operating tasks of the power converter.

Tasks	Power Converter Conditions	Type of Motor
Task A	Inverter	AC Induction Motor
Task B	Rectifier	AC Induction Motor
Task C	Inverter	PMSM
Task D	Rectifier	PMSM

**Table 2:** Component parameters of the simulation circuit.

Symbol	Explanation	Value
*V_D_*	DC source	800 V
*R*_1_	Bleeder resistor	10^−6^ Ω
*R*_2_	Damping resistor	3 Ω
*C*_1_	DC-link capacitor	5.6 × 10^−3^ F
*C*_2_	Damping Capacitor	10^−4^ F
*L*_1_	Converter-side inductor	8 × 10^−4^ H
*L*_2_	Grid-side inductor	2 × 10^−4^ H

**Table 3:** Labeling of power converter OC faults under different tasks.

OC Fault Device		Normal	*S*_1_	*S*_2_	*S*_3_	*S_4_*	*S_5_*	*S_6_*	*S*_1_·*S*_2_	*S*_1_·*S*_3_	*S*_1_·*S*_4_	*S*_1_·*S*_5_
Fault Label	Task A	1	2	3	4	5	6	7	8	9	10	11
	Task B		23	24	25	26	27	28	29	30	31	32
	Task C		44	45	46	47	48	49	50	51	52	53
	Task D		65	66	67	68	69	70	71	72	73	74
**OC Fault Device**		***S*_1_·*S*_6_**	***S*_2_·*S*_3_**	***S*_2_·*S*_4_**	***S*_2_·*S*_5_**	***S*_2_·*S*_6_**	***S*_3_·*S*_4_**	***S*_3_·*S*_5_**	***S*_3_·*S*_6_**	***S*_4_·*S*_5_**	***S*_4_·*S*_6_**	***S*_5_·*S*_6_**
Fault Label	Task A	12	13	14	15	16	17	18	19	20	21	22
	Task B	33	34	35	36	37	38	39	40	41	42	43
	Task C	54	55	56	57	58	59	60	61	62	63	64
	Task D	75	76	77	78	79	80	81	82	83	84	85

**Table 4:** Optimal hyperparameters.

Hyperparameter		Explanation	Best Value
Epoch	Stage I	Training epoch	25
	Stage II		40
	Stage III		90
	Stage IV		170
*μ*		Learning rate	10^−4^
*α*		Fractional order	1.2
*β*		Weight parameter in knowledge distillation	0.5
*T*		Temperature coefficient in knowledge distillation	5
Loss	*L_CE_*	Loss function	Cross entropy
	*L_KL_*		Kullback–Leibler divergence
*λ*		Regularization coefficient	0.05
Batch_size		Batch size	64
Replay_batch_size		Replay batch size	16

**Table 5:** Main parameters of double closed-loop control based on *dq* rotating coordinate system.

Parameters	Explanation	Value
*K_p_*_1_	Voltage outer ring scale factor	0.5
*K_p_*_2_	Current inner loop scaling factor	30
*K_i_*_1_	Voltage outer loop integration factor	210
*K_i_*_2_	Current inner loop integration factor	510
V_DC_^*^	DC Voltage Reference	800
i_q_^*^	Reactive current reference value	0

**Table 6:** The average accuracy, precision, recall, and f1 score of various models across ten predictions under four tasks.

Model	Task A			Task B					Task C				Task D			
	Acc.	Pre.	Rec.	F1.	Acc.	Pre.	Rec.	F1.	Acc.	Pre.	Rec.	F1.	Acc.	Pre.	Rec.	F1.
LL-FO-CE	**0.929**	**0.935**	**0.931**	**0.933**	**0.945**	**0.815**	**0.807**	**0.811**	**0.879**	**0.847**	**0.806**	**0.827**	**0.985**	**0.986**	**0.985**	**0.985**
CNN-Transformer	0.043	0.008	0.025	0.012	0.044	0.024	0.023	0.024	0.234	0.184	0.224	0.154	0.970	0.971	0.970	0.971
Res-BiLSTM	0.045	0.002	0.031	0.003	0.044	0.022	0.023	0.023	0.044	0.002	0.045	0.004	0.971	0.980	0.971	0.976
ASLSTM	0.024	0.001	0.019	0.003	0.015	0.015	0.008	0.011	0.045	0.002	0.04	0.004	0.943	0.921	0.908	0.908
TCN	0.045	0.003	0.028	0.006	0.045	0.016	0.024	0.019	0.045	0.002	0.033	0.003	0.929	0.923	0.924	0.922

## Data Availability

The data presented in this study are available on request from the corresponding author.
